# 747 Burn Mortality Prediction Model and Communication Tool for Healthcare Providers

**DOI:** 10.1093/jbcr/irad045.222

**Published:** 2023-05-15

**Authors:** Harpreet Pangli, Karanvir Raman, Anthony Papp

**Affiliations:** University of British Columbia, Burnaby, British Columbia; University of British Columbia, Vancouver, British Columbia; University of British Columbia, Vancouver, British Columbia; University of British Columbia, Burnaby, British Columbia; University of British Columbia, Vancouver, British Columbia; University of British Columbia, Vancouver, British Columbia; University of British Columbia, Burnaby, British Columbia; University of British Columbia, Vancouver, British Columbia; University of British Columbia, Vancouver, British Columbia

## Abstract

**Introduction:**

Communication is a key competency – the effective exchange of information is essential to a physician’s role. Currently, our centre does not have a communication tool to help guide point of care discussions between healthcare providers and during family meetings. Prognostic relationship of the BAUX score and ABSI index should be determined. An objective communication tool that shows predicted mortality, length of stay (LOS), and number of operations, specifically in our hospital using BAUX index and ABSI score will allow patients and healthcare providers to better understand prognosis, course in hospital, and develop appropriate expectations for outcomes in our centre.

**Methods:**

In this cross-sectional study, all burn patients admitted to our Centre from 2012 to June 2022 were retrospectively recruited. Our burn registry was used to extract complete data of patient information including age, gender, %TBSA, burn depth, presence of inhalational injury, need for ventilator support, intensive care unit (ICU) admission days, hospital LOS, BAUX score, rBAUX score, and ABSI index. Patients were divided into three cohorts: all patients without an inhalation injury, a subgroup of smokers with and without an inhalation injury, and all patients with an inhalation injury. For each cohort, a mean LOS in hospital and/or ICU, number of burn operations, and mortality rate per incremental BAUX score and ABSI index was computed.

**Results:**

A total of 839 patients were included, 725 without and 114 (13.6%) with an inhalation injury. A subgroup of patients (n=286) smokers with and without inhalational injury were separately analyzed. With severity of burn and presence of inhalational injury, both BAUX score and ABSI index show an incremental increase in LOS in hospital, number of operations, and increased mortality. Smokers with rBAUX ≥110 had the longest LOS in hospital, 78.4 hospital days and 35 ICU days. Interestingly, all patients with ABSI 6-7 had the longest LOS/%TBSA and those patients who additionally had an inhalational injury had the highest LOS/%TBSA (5.1 days/%TBSA). Increase in BAUX and ABSI did not correlate with increase in LOS/%TBSA. For BAUX ≥ 90 and ABSI ≥ 8, number of operations and mortality exponentially increased.

**Conclusions:**

Patients with BAUX ≥ 90 and ABSI ≥ 8 should be counselled on a complex course in hospital. Higher BAUX and ABSI correlate with increased mean LOS and number of operations but not LOS/%TBSA. Using our Centre’s burn registry, we can predict course in hospital and provide this to patients, family, and healthcare providers before tertiary centre transfer and admission.

**Applicability of Research to Practice:**

An objective communication model showing the burn centre’s mean LOS, LOS/%TBSA, number of operations, and risk of mortality, can help guide physician-patient and physician-healthcare provider communication. This tool will provide realistic burn recovery expectations at admission, family meetings, and when discussing consent.

